# Coexistence of Diffuse Large B-Cell Lymphoma With Chronic Tubulointerstitial Nephritis: A Case Review and Pathophysiology

**DOI:** 10.7759/cureus.51595

**Published:** 2024-01-03

**Authors:** Kareem Zuhdi, Katsiaryna Khatskevich, Ellen C Riemer

**Affiliations:** 1 Department of Education, University of South Florida (USF) Health Morsani College of Medicine, Tampa, USA; 2 Department of Pathology and Laboratory Medicine, Medical University of South Carolina, Charleston, USA

**Keywords:** diffuse large b cell lymphoma (dlbcl), end-stage renal failure, pathological autopsy, chronic tubulointerstitial nephritis, end-stage renal disease (esrd)

## Abstract

There is an association between lymphomas and kidney disease with renal abnormalities found both in patients with direct infiltration by lymphoma as well as in patients without gross or microscopic evidence of renal involvement. Multiple mechanisms to explain the link between lymphomas and renal disease have been proposed, ranging from direct renal metastasis by the lymphoma to chemokine signaling pathways. In addition, there is a correlation between certain genetic mutations and an increased risk of lymphoma metastasizing to other organs. We present a case of a 41-year-old male who passed away due to end-stage kidney disease and was found on autopsy to have chronic tubulointerstitial nephritis and diffuse large B-cell lymphoma (DLBCL) without direct renal involvement by the lymphoma. The patient had been previously healthy with no significant prior medical history, NSAID, or other contributory medication use of note with the only presenting symptom being renal failure. Only upon autopsy was DLBCL discovered throughout the abdomen with no direct lymphoma involvement evident in the kidneys. To the author’s knowledge, this is one of the few reported cases of DLBCL in English literature without renal infiltration in which the presenting symptom and cause of death was renal dysfunction. Several mechanisms have been theorized for how lymphomas can lead to kidney damage without direct metastasizes; however, more research still needs to be done to better understand the underlying etiology. Given the rarity and the lack of direct infiltration of lymphoma into the kidneys in this patient, we hope reporting this case will allow further advancements in this field of study as well as more comprehensive management.

## Introduction

The relationship of lymphomas with end-stage kidney disease has been reported in the literature to a limited extent, both with and without direct renal metastasis [1]. However, a scarcity of literature has been found with regard to the specific association of diffuse large B-cell lymphoma (DLBCL) with chronic tubulointerstitial nephritis, which we present in this case.

## Case presentation

**Figure 1:**
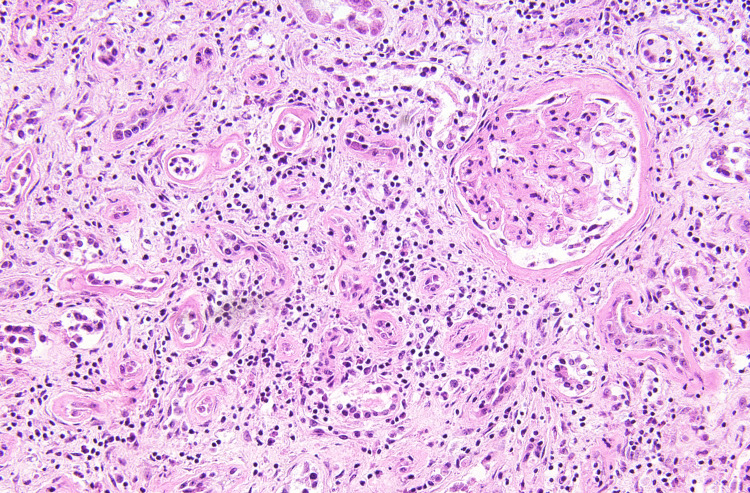
Histology of a kidney section A section of the kidney shows the presence of fibrosis and inflammation consistent with chronic tubulointerstitial nephritis (H&E, 200X).

**Figure 2:**
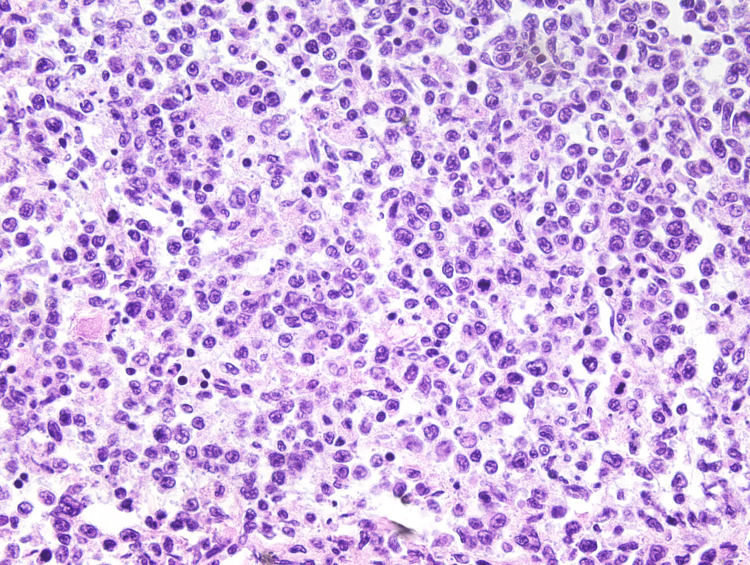
Histology of an abdominal lymph node section An abdominal lymph node section showing the presence of enlarged, atypical lymphocytes with multiple mitotic figures (H&E, 400X).

The patient was a 41-year-old previously healthy male with a recent history of diarrhea, chills, weakness, fatigue, and shortness of breath. He passed away due to end-stage kidney disease, with post-mortem vitreous examination showing an estimated GFR of 11 ml/min, urea nitrogen of 75 mg/dL, and creatinine of 6.3 mg/dL. Further electrolyte examination found a vitreous sodium of 128 mmol/L, potassium of 23.7 mmol/L, chloride of 107 mmol/L, and glucose of 12.0 mg/dl. Findings at autopsy included firm tan kidneys, several pale enlarged abdominal lymph nodes (up to 4.3 cm), pulmonary edema, and pericardial serous effusion (20 mL). The right kidney weighed 190 g (reference range 81-160 g) and the left kidney 230 g (reference range: 83-176 g) [2]. Serial sectioning of the kidneys revealed unremarkable renal parenchyma, vessels, and ureters; no masses or lesions were identified. Upon microscopic examination, chronic tubulointerstitial nephritis was identified with mononuclear inflammation, interstitial fibrosis, and atrophic renal tubules (Figure 1). Immunohistochemistry of the abdominal lymph nodes revealed CD20+ CD34- cells, diagnostic for DLBCL. The unusual component of this case is that the DLBCL was not found to be directly infiltrating the kidneys either grossly or microscopically (Figure 2).

## Discussion

DLBCL is characterized by the proliferation of immunological B cells in lymph nodes and/or other extra-nodal areas throughout the body. DLBCL is the most common type of non-Hodgkin’s lymphoma. While often asymptomatic or underreported, postmortem studies on renal involvement in DLBCL have been shown to be around 10-20% [3,4]. However, limited research has been reported with regard to the specific underlying pathophysiology behind this renal involvement. Several reasons may exist for the lack of association between DLBCL and chronic tubulointerstitial nephritis in the literature. Underreporting and the rarity of the case are very probable reasons. Additionally, this association may have yet not been discerned given many patients with DLBLC may already have comorbidities, chemotherapy regimens, or other medication use that would more likely explain the kidney manifestations. Our case is unique with the lack of significant medical history, medication use, or other more telling causes for the interstitial nephritis. While limited in the generalizability of our findings given the nature of this case being a case study, we hope this work will allow other clinicians to better identify an association between DLBCL and chronic tubulointerstitial nephritis if one exists.

Multiple mechanisms have been theorized about the association between lymphomas and renal involvement. While no cases of DLBCL and chronic tubulointerstitial nephritis were found in the literature at the time of this writing, other lymphomas and renal manifestations can be assessed. A case of acute tubulointerstitial nephritis was found in the literature as the presenting symptom of DLBCL and hypothesized to be a result of direct renal parenchymal infiltration by lymphocytes through undetermined mechanisms [5]. In another study assessing the prevalence of renal involvement in an autopsy series, lymphomatous infiltration was found in 34% of lymphomas while other manifestations such as minimal change nephrosis, monoclonal immunoglobulin deposition amyloidosis, immunotactoid glomerulopathy, and membranous glomerulopathy were found to be much more rare [6]. Chronic tubulointerstitial nephritis is often caused by certain chemotherapeutics; however, in our case, the patient had not undergone any treatment. Other causes of chronic tubulointerstitial nephritis, such as antibiotics, NSAIDs, lithium, or obstructive uropathies, were not involved with our patient as well [1]. The increased weight of the kidneys, as compared to the reference range, maybe another factor in end-stage kidney disease with increased pressure being placed on the nephron [5].

Fourteen immune-related genes (IRGs), AQP9, LMBR1L, FGF20, TANK, CRP, ORM1, JAK1, BACH1, MTCP1, IFITM1, TNFSF10, FGF12, RFX5, and LAP3, were all cited to play a part in DLBCL. These genes were associated with various pathways and pathologies including but not limited to chemokine, toll-like receptor, NK cell signaling, myocarditis, and apoptosis pathways. Expression of these genes was indicated with onset, progression, severity, and spread to other organs [7]. Renal involvement was associated with worse progression of lymphomas [8]. New research is being conducted to assess immunotherapy and potential gene targeting to be able to better understand each unique patient’s pathophysiology and allow for a more focused treatment plan [9]. New guidelines should also consider taking into account renal involvement in their lymphoma workup due to the ever-increasing association being studied.

## Conclusions

We presented a case of a patient who passed away due to end-stage kidney disease with concurrent DLBCL; however, no gross involvement of the kidneys by the DLBCL, raising the question of how the lymphoma may have physiologically affected the kidneys without direct extension of the malignancy. Overall, more research still needs to be done to better understand the mechanism behind lymphoma and its renal involvement. A better understanding of the immune and genetic pathophysiology can potentially guide therapy in the future for DLBCL and create a better assessment for risk stratification and management of less common manifestations. Further research into the link between lymphomas and kidney function can also impact diagnostic and treatment strategies for lymphoma patients with kidney manifestations.
